# Effect of the shape of flapping airfoils on aerodynamic forces

**DOI:** 10.1016/j.heliyon.2024.e29561

**Published:** 2024-04-15

**Authors:** Fahad Butt, Tariq Talha, Rehan Khan, Abdur Rehman Mazhar, Mahad Butt, Jana Petru, Asiful H. Seikh

**Affiliations:** a Digital Pakistan Lab, National University of Sciences and Technology, Islamabad, Pakistan; b Department of Mechanical Engineering, College of Electrical & Mechanical Engineering, National University of Sciences and Technology, Islamabad, Pakistan; c Department of Mechanical Engineering, Ghulam Ishaq Khan Institute of Engineering Sciences and Technology, Topi, Pakistan; d Department of Machining, Assembly and Engineering Metrology, Mechanical Engineering Faculty, VŠB-Technical University of Ostrava, 17. listopadu 2172/15, 708 00, Ostrava, Czech Republic; e Mechanical Engineering Department, College of Engineering, King Saud University, Riyadh, 11421, Saudi Arabia

## Abstract

The rapid exhaustion of fossil fuels and the ozone depletion caused by the excessive usage of the fossil fuels has prompted researchers to look towards bioinspired designs for both propulsion and energy extraction purposes. Limited amount of work has been done to present the effects of airfoil shape on the aerodynamic forces on flapping foils. In this paper, we examine in detail the effect of airfoil camber and its position on flapping foil performance in both energy extraction and propulsion regimes. We also examine the effect of reflex camber on flapping foil performance in both flow regimes. In total, 42 airfoils are analyzed using the NACA 4 and 5-series cross-sections. The man objective of this research is to identify a trend, between airfoil shape and aerodynamic forces. The database created as a result will be used in the future work for designing a hydrokinetic turbine and a bio-inspired unmanned aerial vehicle. The results from the numerical simulations indicate that the airfoil shape has significant effects on the time averaged drag force on the airfoil in both flow regimes. However, the time averaged lift force remains negligible for all cases.

## Nomenclature

c: Airfoil chord length [m]
L: Lift Force [N]
D: Drag force [N]
R: Resultant force [N]
t: instantaneous time [s]
T: period of oscillation [s]
f: Frequency of oscillation [Hz]
H_O_: Heaving amplitude [m]
h(t): Airfoil heave position at time instance t [m]
d: Region of motion for airfoil [m]
U_∞_: Free stream velocity [m/s]
θ(t): Airfoil pitch position at time instance t [rad]
θ_O_: Pitch amplitude [rad]
φ: Phase angle [rad]
γ: Angular frequency [Hz]
θ_m_: Mean pitch angle [rad]
k_1_: Reduced frequency (propulsion) fc/U_∞_
k_2_: Reduced frequency (energy extraction) 2πfc/U_∞_
X_p_: Pivot point
ρ: Fluid density [Kg/m^3^]
$\vec{v}$: Velocity vector
S_m_: Mass source
p: Static pressure
$\overset{═}{\tau }$: Shear stress tensor
$\vec{g}$: Gravity [m/s^2^]
$\vec{F}$: External force [N]
$K_{eff}$: Effective Conductivity $K+K_{t}$
K: Thermal conductivity
$K_{t}$: Turbulent thermal conductivity
$J_{j}$: Diffusion flux of species j
$S_{h}$: Heat of chemical reactions and other heat sources
C_d, inst_: Coefficient of drag, instantaneous
C_d, avg_: Coefficient of drag, averaged over T
C_l, inst_: Coefficient of lift, instantaneous
C_l, avg_: Coefficient of lift, averaged over T
UDF: user-defined function
CFD: Computational Fluid Dynamics
SALSA: Strain-Adaptive Linear Spalart-Allmaras

## 1 Introduction

A flapping mechanism combines the lift and thrust production, the two desired forces in aerodynamics. Flapping can be applied in various flow regimes to gain advantage in the overall efficiency of the wings and rotors of flying machines and turbine blades. The motion of birds and fish has been investigated in detail by previous researchers to make locomotion more efficient [[1], [2], [3], [4], [5], [6], [7], [8], [9], [10], [11], [12], [13], [14], [15], [16], [17], [18], [19], [20], [21]]. Various researchers have studied the effects of airfoil shape on the aerodynamic performance of airfoils [[10], [11], [12], [13], [14], [15], [16], [17], [18], [19], [20], [21]]. A brief review of the previously published research work is presented in the following paragraphs.

A self-induced oscillating hydrofoil attached to an arm was numerically investigated at Reynolds number of 1100 using a commercial CFD code [10]. The effects of camber were examined along with the effects of arm length and swing direction. In this study, the authors employed a C-shape hydrofoil. The results from the numerical simulations indicated the camber and pitch angle have significant effects on the aerodynamics forces on the airfoil, therefore on the power extraction as well. Quantitatively, a maximum efficiency of the turbine is at 0.285. The authors also studied various configurations of the arm - this portion of the study highlighted that the upwind and camber arm configuration achieves better performance over the downwind arm and camber configurations. An optimal pitch angle was found to be 143° for upwind arm configuration [10].

The effects of large amplitude and non-sinusoidal motion on airfoil undergoing pitching motion was numerically analyzed using a commercially available code [11]. The airflow was subjected to flow which corresponds to the propulsion regime. NACA 0012 airfoil was selected for this part of study. To study the effects of camber on airfoil performance, three cambered NACA sections were employed with different camber magnitudes and locations. The Reynolds number for this study was kept constant at 1.35 × 10^4^. This study revealed that adding camber to the airfoils has negligible effect on the thrust coefficient. The results for this study also indicated that the thrust coefficient increase with increase in reduced frequency while keeping the amplitude of oscillation fixed [11].

The effect of airfoil thickness and camber on airfoil propulsive performance was analyzed numerically at Reynolds number in the range of 200 and 2.0 × 10^6^ [12]. To study the effect of thickness on airfoil performance, the NACA 4-series symmetric airfoils with thickness varying from 0.06c-0.5c were selected. The effect of camber was investigated using NACA 4-series airfoils with different camber magnitude and location. The airfoils considered include NACA 4215, 4415 and 4615. The results of this study revealed that adding camber to the airfoil shows a very small improvement in terms of the thrust coefficient in comparison to that of symmetric airfoils. It was also shown in this study that the thin airfoils outperform the thick airfoils at various Reynolds numbers [12].

In a relatively recent study, various types of airfoils including symmetric airfoils with varying thickness and maximum thickness locations; cambered airfoils with same thickness, thickness position and camber but different maximum camber positions; and cambered airfoils with same thickness, position and camber position but different maximum camber were analyzed [13]. The airfoils investigated were NACA 4 and 6-series cross-sections. It was found in this study that efficiency of the hydrofoil first increases and subsequently decreases with an increase in maximum thickness while keeping maximum camber location constant. Furthermore, efficiency of the hydrofoil was found to increase and then decrease afterwards when the position of maximum thickness moves from the leading-edge towards the trailing-edge. It was also found that the efficiency for cambered airfoils with same thickness is lower than the airfoils with larger camber [13].

The effects of camber angle on the aerodynamic performance of a flapping wing micro air vehicle is examined in a recently published work [14]. The effects of varying camber angle and flapping frequency were analyzed. It was found that the vortex structures are significantly affected by the frequency and magnitude of the twisting motion of the wing. The frequency and magnitude were found to be dependent on the camber angle. Quantitatively, on the recommended operating frequency of 24 Hz, a camber angle of 15° has the highest propulsive efficiency. The propulsive efficiency was found to be 14 % more than the non-cambered wing [14]. The maximum camber was varied from 0.0c to 0.3c to investigate the effect of maximum camber on lift for a flat plate [15]. Numerical simulations were employed to study the lift characteristics of the wing. To change the camber of the wing, the chordwise curvature was applied to the cross-section, thereby resulting in various different wing shapes. It was found that the wings having higher camber result in flapping foil generating more lift. It was also reported that the most averaged lift is generated at the location of maximum camber which is nearest to the center of the mean average chord [15].

The aerodynamic effects of changes in the shape of a wing of a hovering hawkmoth were studied in detail [16]. The wing shape was controlled by varying the twist, the camber and the spanwise bending. It was found that introducing curvature in the wing can increase the aerodynamic force with negligible effects on the aerodynamic performance. An optimum wing shape was found to increase lift or decrease drag by using numerical simulations [16]. The effects of changing airfoil camber and wing twist investigated numerically [17]. It was found that the aerodynamic forces on the flapping wing were more effected by the changing camber as compared to the wing twist. The wing deformation was shown to increase the maximum lift coefficient. Furthermore, it was suggested that the wing deformation can also result in the reduction of the power requirement for flight [17].

Four different cambers were created to study the effect of camber on the aerodynamic properties of airfoils [18]. The cambers range from 0.125c to 0.25c, with increments of 0.042c. The study considered flat-plate based airfoils. The numerical simulations performed as a part of this study revealed an optimum camber of 0.167c to have the highest average lift and thrust values. The effect of camber on the lift was found to be more prominent than the effect of camber on the thrust produced by the airfoils [18]. Various parameters of wings of micro-air vehicles such as airfoil camber, twist angle, and aspect ratio etc. were analyzed using CFD [19]. The study also utilized flat-plate airfoils [19] with maximum camber and its location ranging from 0.05c – 0.2c and 0.25c – 0.75c. It was found that the amount of camber significantly changes the resultant aerodynamic force direction. Furthermore, the change in the location of the maximum camber was found to have less significant impact on either the lift coefficient or the rotary moment coefficient [19].

The effects of spanwise twist and chordwise camber for micro-air vehicles were studied in detail using numerical simulations [20]. Quantitatively, the results indicated that the amount of camber plays a significant role in the improvement of both lift and L/D. An optimum configuration was found to improve lift force and L/D by up to 16.7 and 10.6 %, respectively. An experimental investigation was performed for flapping wings with and without camber to study the effect of camber amount on wing performance [21]. The main goal of conducting the experiments is to study the aerodynamic effect of cambered wings for flapping-wing micro air vehicles. The study was conducted at a Reynolds number of 3600. For the amounts of cambers analyzed in this study, the C_l, avg_ was reported to increase as the amount of camber increases while the C_d, avg_ remained largely unchanged as compared to the flat wing [21].

The study [26] introduced a reactive control strategy using a Reinforcement Learning (RL) to govern the motion of a rotating flap on a NACA0012 airfoil for aerodynamic performance enhancement. CFD simulations were used to build data set, and Artificial Neural Networks (ANNs) model aerodynamic coefficients. The trained RL agent effectively adapts to different angles of attack (AoA), achieving nearly optimal results. Rapid and accurate responses were observed for step, ramp, and random signals, demonstrating the neural network's versatility. The study was conducted at a Reynolds number of 1.9 × 10^6^. It is interesting to note neural networks being used for aerodynamic optimization [26]. A research work compared the effect of various turbulence models on flapping foil. By comparing the results with both known experimental and calculated data, it was demonstrated that the flows having developed and significant flow separation, which are characterized by unsteady effects in turbulence, the SALSA turbulence model exhibited higher performance than the other tested models. By comparing the results with the established experimental data, the aerodynamic coefficients obtained with the SALSA turbulence model were found to be 10–15 % more accurate than those obtained with other turbulence models [27].

It can be clearly seen from the literature review presented that the airfoils selection is generally random when it comes to studies concerning the airfoil shape in flapping motion. A limited range of airfoil cambers and the location of maximum camber was investigated [[10], [11], [12], [13], [14], [15], [16], [17], [18], [19], [20], [21]]. Therefore, a more methodological and rigorous design space of 42 airfoils is created as a part of the present study, as explained in Section 2, Sub-Section B. Numerical methodology is presented in the “Methodology” section which is followed by the “Results and Discussion” section.

## 2 Methodology

**Fig. 1:**
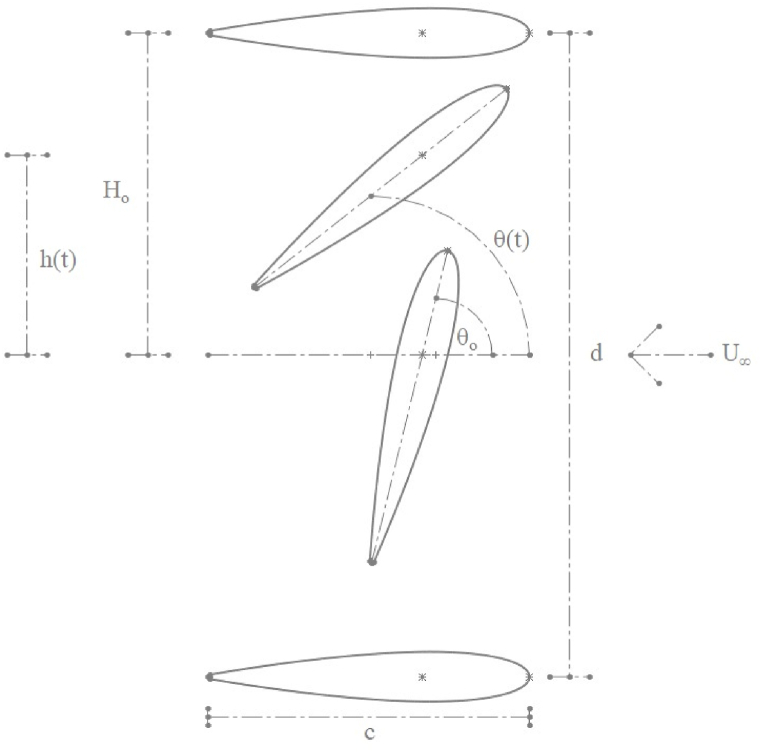
Various locations of airfoil undergoing flapping motion.

**Fig. 2:**
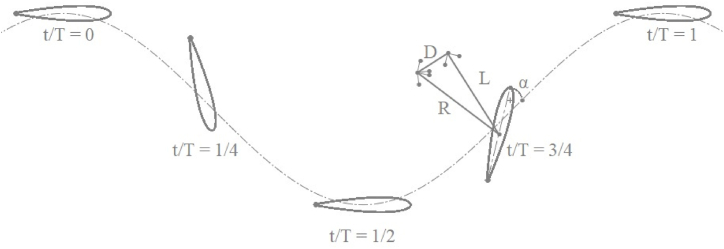
Airfoil motion at various instances in time.

The flapping motion in airfoils is achieved by a combination of heaving and pitching motion. The motion of airfoil is illustrated in Fig. 1, Fig. 2. Within Fig. 2, the airfoil motion is from left to right of the viewer. The airfoil motion is viewed from the reference from of free stream velocity, U_∞_. The governing equation for the airfoil motion in the power extraction regime is given by equations (1), (2)). While equations (3), (4)) present the equations of motion for the airfoil in the propulsion regime. The phase angle is kept at π/2 rad for all the cases. $\vartheta_{m}$ is kept at 0 rad for the power propulsion cases [1]. Air as an ideal gas is considered as a fluid for all the cases studied. The ambient pressure and temperature are kept at 101325 Pa and 293.15 K, respectively. Various parameters used in the propulsion and power extraction cases is mentioned in Table 1.

(1) $$\begin{array}{c} \vartheta \left(t\right)=\vartheta_{O}\;\sin\left(\gamma t\right) \end{array}$$

(2) $$\begin{array}{c} h\left(t\right)=h_{O}\;\sin\;\left(\gamma t+\varphi \right) \end{array}$$

(3) $$\begin{array}{c} \vartheta \left(t\right)=\vartheta_{m}+\vartheta_{O}\;\cos\;\left(\gamma t+\varphi \right) \end{array}$$

(4) $$\begin{array}{c} h\left(t\right)=h_{O}\;\cos\;\left(\gamma t\right) \end{array}$$

**Table 1:** Parameters of flow for each regime.

Parameter	Value	
	Power Extraction	Propulsion
$\vartheta_{O}$	1.3323	0.5236
$h_{O}$	1	1
Re	1100	1000
U_∞_	0.01662	0.01511
k_1_	0.14	N/A
K_2_	N/A	1.41
f	0.002327	0.003391
T	429.721	294.8951
t	T/500	T/500
γ	0.0146	0.02131
X_p_	0.3333c	0.25c

The aerodynamic characteristics of the selected airfoils are analyzed using a commercially available CFD code, ANSYS Fluent [22]. Fluent employs the cell-centered finite volume method. We use the laminar model along with the SIMPLE solution algorithm. The discretization of the continuity, momentum and energy equations (equations (5), (6), (7))) [23] is obtained by using the second-order accurate Upwind scheme. Time is discretized using the implicit second-order accurate scheme. Dynamic mesh with smoothing and remeshing options is used to simulate the flapping motion via “define_cg_motion” UDF [23]. The UDF is coded as part of this research work.

(5) $$\begin{array}{c} \frac{\partial \rho }{\partial t}+\nabla *\left(\rho \vec{v}\right)=S_{m} \end{array}$$

(6) $$\begin{array}{c} \frac{\partial }{\partial t}\left(\rho \vec{v}\right)+\nabla *\left(\rho \vec{v}\vec{v}\right)=-\nabla p+\nabla *\left(\tau \right)+\rho \vec{g}+\vec{F} \end{array}$$

(7) $$\begin{array}{c} \frac{\partial }{\partial t}\left(\rho E\right)+\nabla *\left(\vec{v}\left(\rho E+p\right)=\nabla *(K_{eff}\nabla T-\sum_{j}h_{i}\vec{j_{j}}+\left(\overset{═}{\tau }*\vec{v}\right)\right)+S_{h} \end{array}$$

A. Validation and Verification

The accuracy of the present numerical simulations is ascertained by comparing the results with the published data [1,2]. The data comparison with [1] is for the propulsion regime while the validation for the power extraction regime is done by comparing the present results with [2]. A square computational domain is chosen for the present simulations. The size of the computational domain is kept at 40c in all directions, which is larger in size than the size used in the previously published literature [1,2]. This is done to ensure that the walls of the computational domain have no effect on the simulation results. The mesh and computational domain are presented in Fig. 7 showing localized mesh refinement in various areas of interest.

**Fig. 3:**
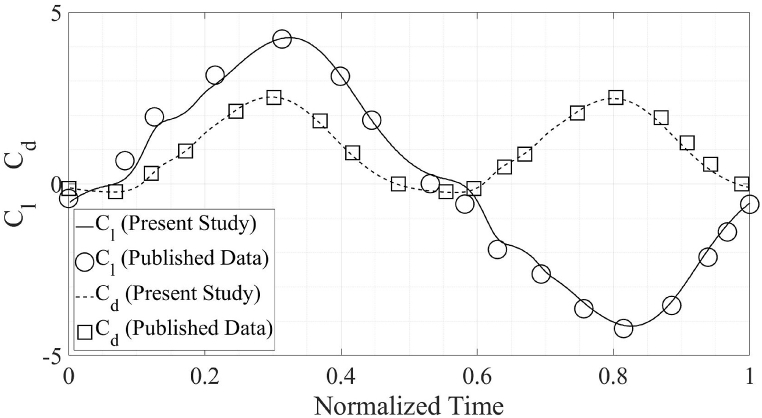
Comparison of the results of present simulations with previously published results, in the propulsion regime [1].

**Fig. 4:**
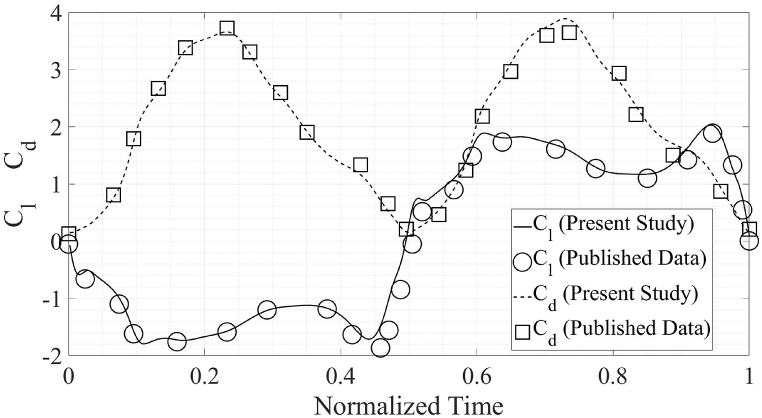
Comparison of the results of present simulations with previously published results, in the energy extraction regime [2].

The C_d, avg_ from the present numerical simulations comes out to be 0.984 as compared to 0.996 from the published results [1], for the propulsion flow regime. The C_d, avg_ from the present numerical simulations is 2.024 while the C_d, avg_ from Ref. [2] is 2.017. Meanwhile, C_l, avg_ from the present simulations is negligible as is the case in published literature [1,2]. The C_l, max_ for energy extraction regime in Ref. [2] is 1.902 in comparison to 2.024 from the present simulations. For the propulsion flow regime, the published C_l, max_ [1] is 4.217 as compared with the C_l, max_ from present simulations 4.148. The comparison of the results from the simulations performed as a part of this study with previously published literature is presented in Fig. 3, Fig. 4. The plots are for one complete flapping cycle.

**Fig. 5:**
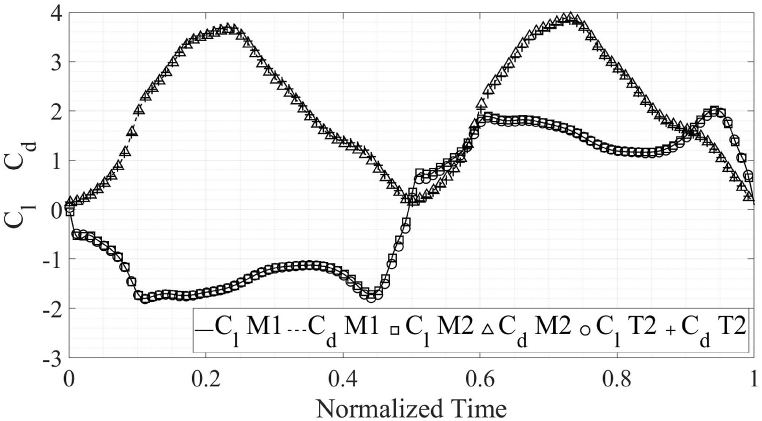
Comparison of the results for various verification cases, energy extraction regime.

**Fig. 6:**
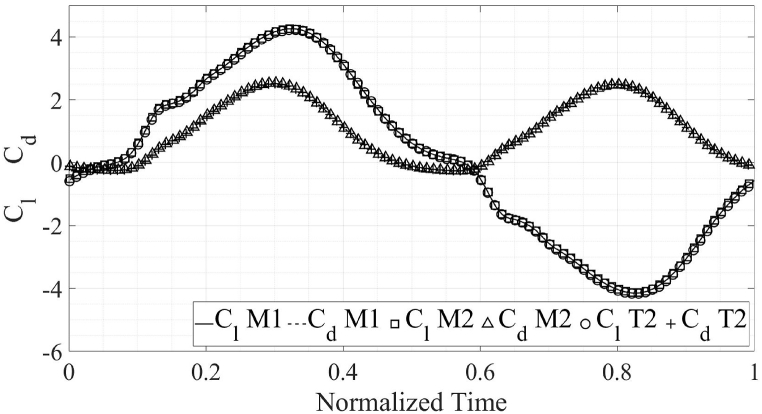
Comparison of the results for various verification cases, propulsion regime.

**Fig. 7:**
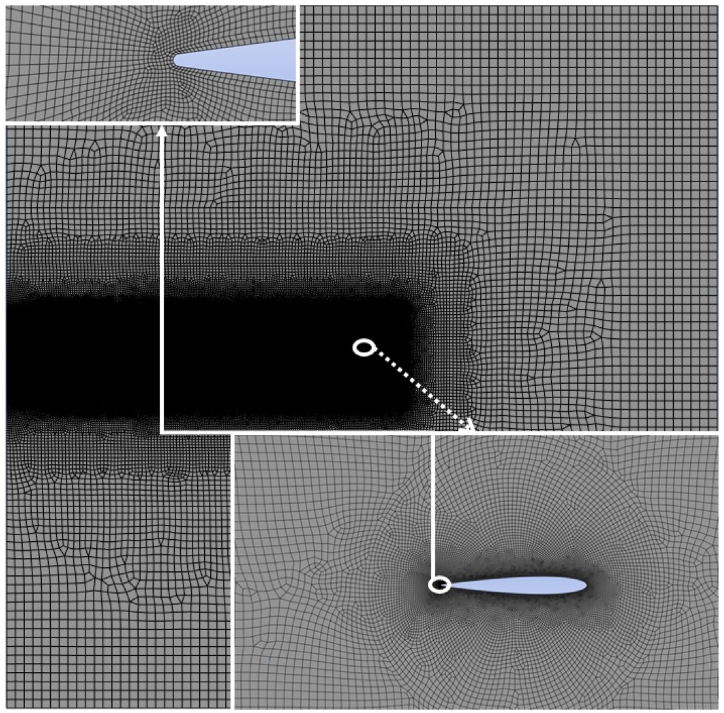
The computational domain and the mesh.

Furthermore, mesh and time-step independence tests are carried out to ensure that the results from the numerical simulations depend on the boundary conditions alone and are not affected by these two properties. The results from the mesh and time-step independence studies are presented in Fig. 5, Fig. 6 for both flow regimes. It is clear that for both flow regimes, decreasing the time-step and cell size do not yield any accuracy improvements over the initial cell and time-step size. Therefore, the initial mesh and time-step size is selected for further simulations performed as a part of this study. It is important to note that the initial mesh and time-step size are used in the validation of our work with previously published data, as explained in Section 2, Sub-Section A. For clarity, only 1/10th of the data points are shown for the Case T1 and M2. The details about the cases M1, M2 and T1 are mentioned in Table 2. For the case M2, the mesh count is increased by 150 % as compared to the case M1. However, the observed difference in the results is only 0.5 %. The case T1 has time step reduced by 50 % as compared to the case M1 with negligible change in the results. It should be noted that local mesh controls are used to refine the mesh in areas of interest i.e. around and in the wake of the airfoil to ensure solution accuracy, as shown in Fig. 7. The mesh, made up of quadrilateral cells. The mesh for the case M1 is shown in Fig. 7. A closeup of the mesh at the trailing-edge and in the wake of the airfoil is also shown within Fig. 7 to clearly show localized grid refinement. Furthermore, 96 % of the cells in the mesh had orthogonal quality of unity.

B. Airfoil Shapes

**Table 2:** Comparison of various cases.

Case	Cell Count		Time-Step	
Regime	Energy Extraction	Propulsion	Energy Extraction	Propulsion
M1	148,188	148,550	T/500th T/500th T/1,000^th^	
M2	213,389	213,966		
T1	148,188	148,550		

To study the effect of airfoil camber, NACA 4-series airfoils are considered. The amount of camber is changed from 0.02c to 0.08c, in increments of 0.02c. The camber position is varied by changing the camber location systematically from 0.2c to 0.8c, in increments of 0.2c. To study the effect of reflex camber on flapping foil performance, NACA 5-series airfoils are considered. The camber position and reflex are increased from 0.1c to 0.25c in increments of 0.05c and the design lift coefficient are changed from 0.2 to 0.8 in increments of 0.2. The details about equations used to plot NACA 4-series airfoils can be found in Ref. [24] while details about the NACA 5-series airfoils are available in Ref. [25].

## 3 Results and Discussion

The results and trends from the numerical simulations are presented and discussed in the following Sub-Sections. The quantitative results are tabulated in Table 3, Table 4, Table 5, Table 6, Table 7, Table 8. It is to be noted that for the airfoils operating in the energy extraction flow regime, the minimum C_l, inst_ occurs near 0.5T (end of downstroke) while the maximum C_l, inst_ exists near 1.0T (end of upstroke). Meanwhile, for the airfoils operating in the propulsion regime, minimum C_l, inst_ occurs near 0.75T (middle of downstroke) and maximum C_l, inst_ exists near 0.25T (middle of upstroke). The maximum C_d, inst_ occurs near 0.25T and 0.75T. The minimum C_d, inst_ exists near 0.0T, 0.5T and 1.0T for both flow regimes.

A. NACA 4-Series Airfoils

**Table 3:** NACA 4-series drag coefficients.

Regime	Energy Extraction			Propulsion		
Airfoil	Maximum C_d, inst_	Minimum C_d, inst_	C_d, avg_	Maximum C_d, inst_	Minimum C_d, inst_	C_d, avg_
2210	4.005	0.096	2.054	0.241	−2.588	**−0.986**
2410	3.981	0.105	2.047	0.212	−2.574	−0.981
2610	3.97	0.1	2.045	0.204	−2.576	−0.979
2810	3.977	0.103	2.048	0.201	−2.565	−0.981
4210	4.003	0.082	2.07	0.303	−2.698	−0.96
4410	3.969	0.135	2.061	0.24	−2.683	−0.951
4610	3.96	0.11	2.059	0.178	−2.658	−0.955
4810	3.969	0.116	2.057	0.177	−2.65	−0.964
6210	3.946	0.105	2.103	0.326	−2.766	−0.927
6410	3.898	0.154	2.069	0.418	−2.738	−0.871
6610	3.883	0.120	2.056	0.231	−2.685	−0.898
6810	3.907	0.135	2.079	0.146	−2.69	−0.93
8210	**4.066**	0.11	**2.154**	0.326	**−2.808**	−0.891
8410	4.055	**0.224**	2.066	**0.437**	−2.673	−0.837
8610	4.053	0.12	2.053	0.358	−2.657	−0.872
8810	4.016	0.151	2.091	0.185	−2.694	−0.887

**Table 4:** NACA 4-series lift coefficients.

Regime	Energy Extraction			Propulsion		
Airfoil	Maximum C_l, inst_	Minimum C_l, inst_	C_l, avg_	Maximum C_l, inst_	Minimum C_l, inst_	C_l, avg_
2210	2.0794	−2.061	0.085	4.195	−4.0351	0.035
2410	1.8888	−2.079	0.051	4.101	−4.0788	−0.029
2610	1.8661	−2.157	0.024	4.079	−4.0788	−0.042
2810	1.8813	−2.121	0.034	4.072	−4.1078	−0.048
4210	1.8867	−2.241	0.049	4.29	−3.9188	0.182
4410	1.8927	−2.343	0.034	4.122	−3.9624	0.1
4610	1.8867	−2.518	0.017	4.042	−4.0424	−0.051
4810	1.8927	−2.392	0.004	4.035	−4.1660	−0.097
6210	**2.2232**	−2.367	**0.172**	4.442	−3.8461	0.231
6410	1.9108	−2.668	0.007	4.13	−3.8534	0.14
6610	1.8927	−2.89	−0.013	3.999	−4.0569	−0.103
6810	1.9168	−2.872	−0.029	3.977	−4.2605	−0.153
8210	2.0430	−2.578	0.138	**4.617**	−3.7516	**0.266**
8410	1.9048	−2.848	−0.012	4.122	−3.9479	−0.081
8610	1.9048	−3.155	−0.034	3.992	−4.195	−0.305
8810	1.9168	**−3.305**	−0.078	3.955	**−4.406**	−0.28

**Table 5:** NACA 5-series without reflex camber drag coefficients.

Regime	Energy Extraction			Propulsion		
Airfoil	Maximum C_d, inst_	Minimum C_d, inst_	C_d, avg_	Maximum C_d, inst_	Minimum C_d, inst_	C_d, avg_
12010	4.047	0.087	2.054	0.215	−2.493	−0.985
13010	4.036	0.088	2.055	0.219	−2.511	−0.987
14010	4.023	0.085	2.054	0.224	−2.528	−0.986
15010	4.004	0.094	2.054	0.227	−2.537	−0.986
22010	4.079	0.085	2.055	0.243	−2.553	−0.983
23010	4.070	0.101	2.062	0.254	−2.583	−0.987
24010	4.050	**0.114**	2.069	0.262	−2.606	**−0.987**
25010	4.016	0.109	2.066	0.253	−2.614	−0.985
42010	4.070	0.092	2.060	0.279	−2.606	−0.978
43010	4.069	0.070	2.065	0.285	−2.641	−0.979
44010	4.058	0.071	2.073	0.289	−2.662	−0.975
45010	4.016	0.076	2.082	0.314	−2.688	−0.963
52010	4.059	0.060	2.061	0.296	−2.639	−0.967
53010	4.039	0.049	2.070	0.307	−2.682	−0.966
54010	**4.089**	0.054	2.087	0.308	−2.707	−0.958
55010	4.048	0.087	**2.112**	**0.317**	**−2.715**	−0.944

**Table 6:** NACA 5-series with reflex camber drag coefficients.

Regime	Energy Extraction			Propulsion		
Airfoil	Maximum C_d, inst_	Minimum C_d, inst_	C_d, avg_	Maximum C_d, inst_	Minimum C_d, inst_	C_d, avg_
12110	4.056	0.085	2.054	0.215	−2.489	−0.986
13110	4.047	0.083	2.055	0.228	−2.510	−0.986
14110	4.027	0.093	2.058	0.230	−2.521	−0.987
15110	4.013	0.097	2.058	0.234	−2.538	−0.987
22110	4.096	0.096	2.060	0.244	−2.544	−0.984
23110	4.104	0.115	2.073	0.260	−2.569	**−0.988**
24110	4.078	0.095	2.074	0.270	−2.592	−0.987
25110	4.040	0.083	2.078	0.278	−2.613	−0.980
42110	4.083	0.090	2.063	0.278	−2.588	−0.976
43110	4.117	0.063	2.071	0.289	−2.610	−0.978
44110	4.176	0.054	2.083	0.300	−2.638	−0.971
45110	4.140	0.062	2.109	**0.310**	−2.649	−0.956
52110	4.071	0.062	2.064	0.298	−2.614	−0.965
53110	4.068	0.039	2.077	0.304	−2.633	−0.963
54110	4.202	0.046	2.113	0.300	**−2.651**	−0.956
55110	**4.275**	**0.171**	**2.189**	0.271	−2.607	−0.937

**Table 7:** NACA 5-series without reflex camber lift coefficients.

Regime	Energy Extraction			Propulsion		
Airfoil	Maximum C_l, inst_	Minimum C_l, inst_	C_l, avg_	Maximum C_l, inst_	Minimum C_l, inst_	C_l, avg_
12010	2.138	−1.864	0.088	4.145	−4.040	0.031
13010	2.142	−1.911	0.088	4.152	−4.053	0.020
14010	2.094	−1.906	0.089	4.165	−4.059	0.018
15010	2.104	−1.943	0.085	4.164	**−4.070**	0.009
22010	2.267	−1.879	0.120	4.238	−3.985	0.086
23010	2.165	−1.930	0.105	4.255	−4.016	0.088
24010	2.173	−1.983	0.110	4.269	−4.012	0.094
25010	2.092	−2.033	0.104	4.233	−4.014	0.086
42010	2.032	−1.978	0.085	4.348	−3.953	0.157
43010	1.871	−2.057	0.057	4.359	−3.966	0.158
44010	1.915	−1.995	0.096	4.354	−3.958	0.171
45010	1.893	**−2.164**	0.072	4.331	−3.919	0.213
52010	1.915	−1.995	0.096	4.444	−3.924	0.203
53010	1.870	−2.086	0.111	**4.490**	−3.923	0.209
54010	2.080	−2.064	0.170	4.476	−3.894	**0.226**
55010	**2.386**	−2.130	**0.212**	4.423	−3.890	0.222

**Table 8:** NACA 5-series with reflex camber lift coefficients.

Regime	Energy Extraction			Propulsion		
Airfoil	Maximum C_l, inst_	Minimum C_l, inst_	C_l, avg_	Maximum C_l, inst_	Minimum C_l, inst_	C_l, avg_
12110	2.161	−1.830	0.096	4.153	−4.041	0.034
13110	2.199	−1.895	0.097	4.176	−4.048	0.036
14110	2.235	−1.899	0.109	4.183	−4.057	0.025
15110	2.179	−1.895	0.105	4.196	**−4.062**	0.024
22110	2.359	−1.850	0.134	4.253	−3.985	0.103
23110	2.165	−1.919	0.095	4.288	−4.004	0.107
24110	1.947	−1.986	0.077	4.296	−4.011	0.117
25110	1.920	−1.936	0.107	4.298	−3.980	0.155
42110	2.048	−1.947	0.088	4.368	−3.951	0.168
43110	1.874	−1.952	0.072	4.405	−3.964	0.175
44110	1.971	−1.901	0.148	4.422	−3.939	0.200
45110	2.323	−1.902	0.221	4.402	−3.909	0.227
52110	2.008	−1.921	0.114	4.402	−3.909	0.227
53110	2.095	**−1.987**	0.153	4.538	−3.921	0.222
54110	2.663	−1.922	**0.244**	**4.555**	−3.906	**0.229**
55110	**2.817**	−1.951	0.214	4.450	−3.916	0.161

The discussion about NACA 4-series airfoils operating in the energy extraction regime is presented first. The maximum C_d, inst_ increases as the amount of camber is increased. The minimum C_d, inst_ also increases as the amount of camber is increased. C_d, avg_ increases with an increase in the amount of maximum camber for airfoils having the location of maximum camber at the extreme ends of the airfoils i.e. near leading and trailing-edges. Meanwhile, C_d, avg_ increases then subsequently decreases afterwards as the amount of camber is increased for the airfoils having maximum camber location near the middle of the chord length of the airfoils. The maximum C_d, inst_ increases as the camber position is moved towards the leading-edge of the airfoils. The minimum C_d, inst_ increases as the location of the maximum camber is moved away from the leading-edge of the airfoil. C_d, avg_ increases as the camber location is moved towards the leading-edge of the airfoils.

The maximum C_l, inst_ increases as the amount of maximum camber increases while the minimum C_l, inst_ shows an opposite trend. The maximum C_l, inst_ increases as the location of maximum camber is moved towards the leading-edge of the airfoil. The minimum C_l, inst_ increases as the location of the maximum camber is moved towards the leading-edge of the airfoil. C_l, avg_ remains zero for all the cases while the airfoil is in the energy extraction flow regime.

**Fig. 8:**
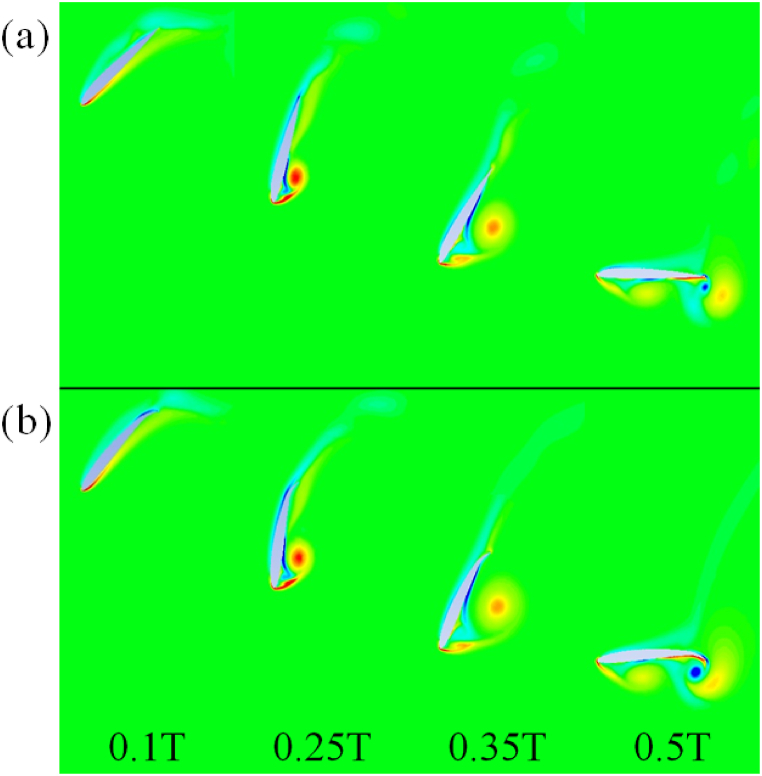
Airfoils undergoing downstroke motion. (a) NACA 2210, (b) NACA 8810.

**Fig. 9:**
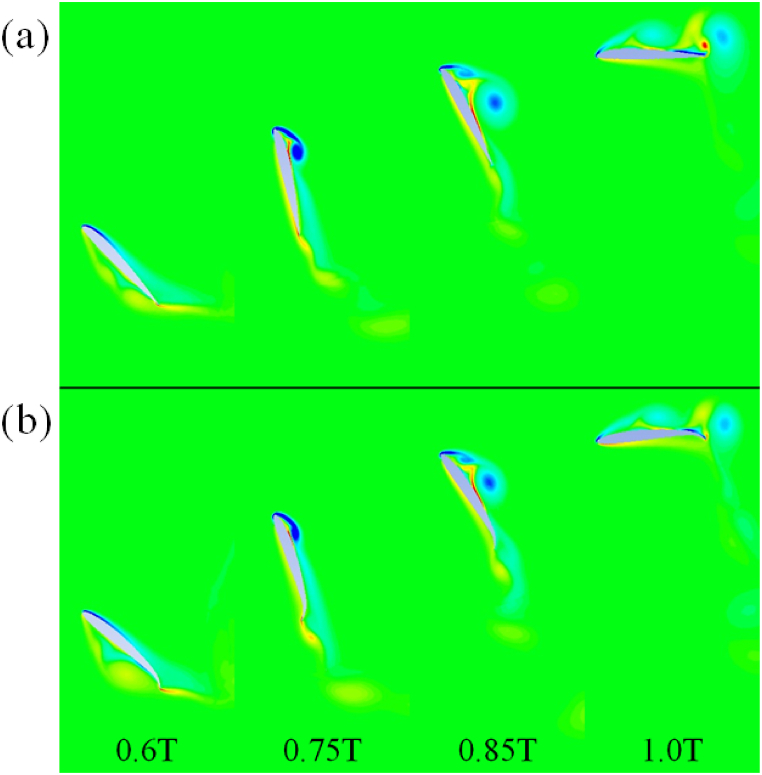
Airfoils undergoing upstroke motion. (a) NACA 2210, (b) NACA 8810.

The post-processed data from the NACA 2210 and 8810 airfoils is used to explain the change in C_d_ and C_l_ due to variations in the maximum camber and maximum camber location as shown in Fig. 8(a) and (b). The magnitude of vorticity around the axis perpendicular to both C_l_ and C_d_ during the downstroke and upstroke is shown in Fig. 8, Fig. 9. An anticlockwise vortex is observed at the leading-edge of the two airfoils during downstroke and vice-versa. A vortex forming at the trailing-edge with opposite direction of rotation to the leading-edge vortex is also observed as shown in Fig. 9(a) and (b). The trailing-edge vortex is stronger in magnitude for airfoils with high camber and the location of camber near the trailing-edge of the airfoils during the downstroke and vice-versa.

**Fig. 10:**
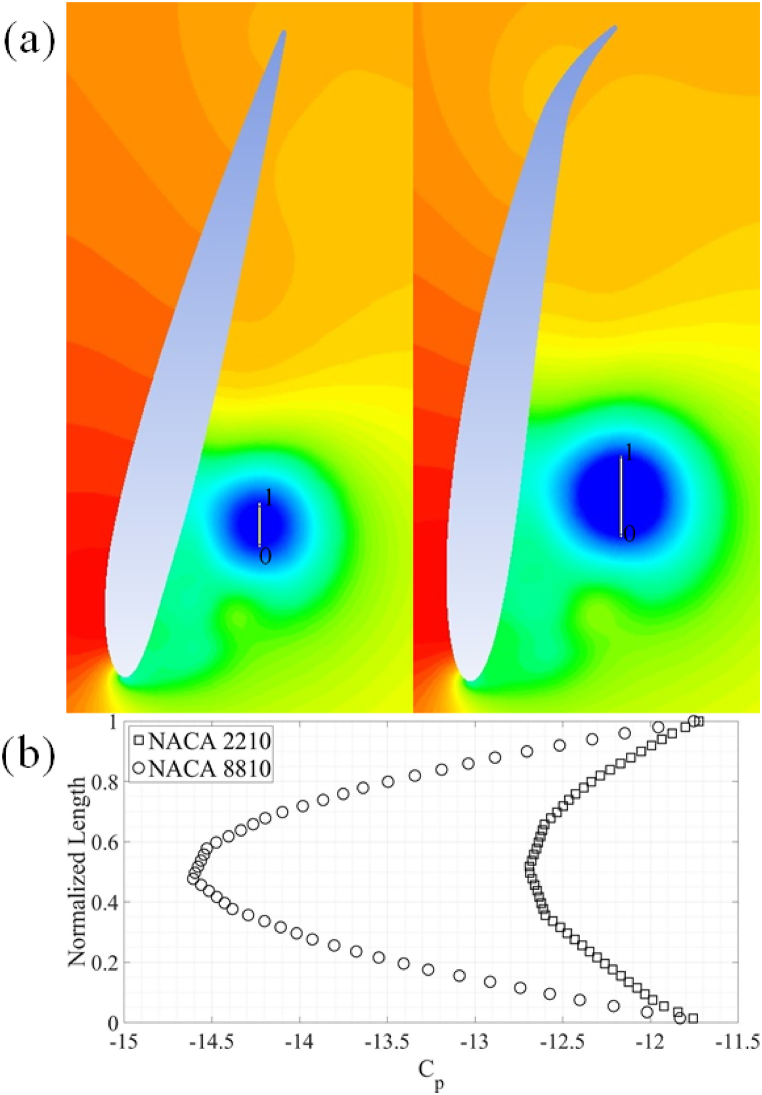
(a) NACA 2210 and NACA 8810 airfoils at 0.25T during downstroke motion. (b) Comparison of C_p_.

This vortex formation leads to the formation of corresponding zones of low pressure in the wake of the airfoils, resulting in a higher C_d, avg_ for airfoils with high camber and maximum camber location near the trailing-edge. The low-pressure regions in the wake of airfoils is clearly visible in Fig. 10(a) and (b). The C_p_ is plotted along the yellow line which is visible in the wake of airfoils. It should be noted that the diameter of the vortex for NACA 8810 airfoil is 0.12c while the diameter of the vortex in the wake of NACA 2210 comes out to be 0.069c. In terms of C_l, avg_, it is observed that the gain in C_l, avg_ for upstroke due to the leading-edge vortex is canceled out by additional negative lift generated during the downstroke. Hence, the C_l, avg_ for the complete cycle remains zero.

For the NACA 4-series airfoils operating in the propulsion regime, C_d, avg_ increases as the amount of maximum camber decreases. The minimum C_d, inst_ increases as the amount of camber increases. C_d, avg_ at first decreases then increases as the location of the maximum camber is moved towards the leading-edge of the airfoil. The minimum C_d, inst_ increases as the location of maximum camber is moved towards the leading-edge of the airfoil. The maximum C_d, inst_ remains negligible for all cases.

If the location of the maximum camber is moved towards the leading-edge of the airfoil, the maximum C_l, inst_ increases. The minimum C_l, inst_ also increases as the location of the maximum camber is moved towards the leading-edge. A reduction in the amount of maximum camber causes the maximum C_l, inst_ to increase for the cases when the maximum camber location is far away from the leading-edge and decreases when the location of the maximum camber is near the leading-edge of the airfoil. The minimum C_l, inst_ increases as the amount of maximum camber decreases when location of maximum camber is far away from the leading-edge of the airfoil. The minimum C_l, inst_ increases with increase in maximum camber when location of maximum camber is near the leading-edge of the airfoil. C_l, avg_ remains negligible for all the cases investigated.

**Fig. 11:**
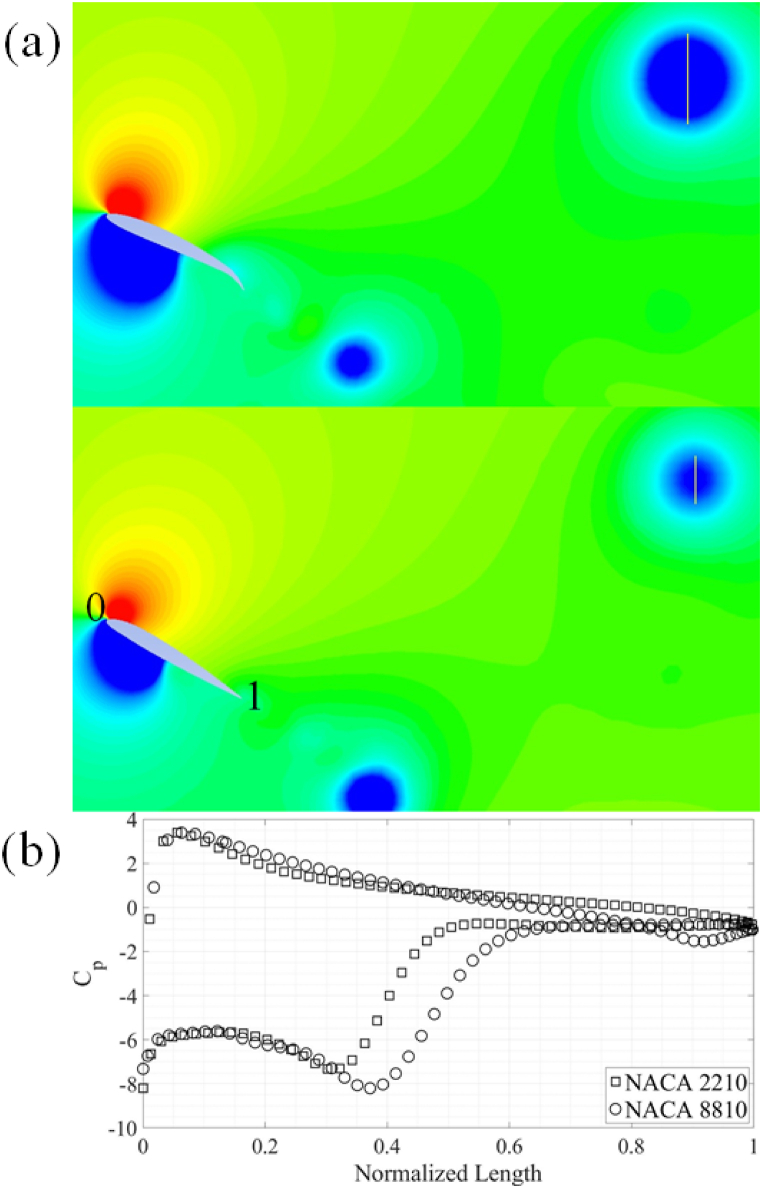
(a) NACA 2210 and NACA 8810 airfoils at 0.25T during upstroke motion. (b) Comparison of C_p_.

As was the case with the energy extraction regime for 4-series NACA airfoils, NACA 8810 and 2210 airfoils are employed to further explain the results. A vortex of higher strength and size is formed at the leading-edge of the airfoils with large camber and the location of maximum camber near the leading-edge of the airfoils. This vortex after being shed, leads to zones of relatively lower pressure in wake of the airfoils with high camber with location of maximum camber located near the trailing-edge of the airfoils. This particular vortex dynamics leads to more drag being produced by airfoils with less maximum camber and the location of maximum camber near the leading-edge of airfoils. The pressure distribution around and in the wake of the airfoils in shown in Fig. 11(a). The C_p_ values shown in Fig. 11(b) are extracted along surface of the airfoils. The diameter of the vortex for NACA 2210 airfoil is observed to be 0.31c while the vortex diameter for the NACA 8810 is 0.58c. The diameter is clearly indicated by the yellow lines visible towards the top right corner of Fig. 11.

As is observed in the case of the energy extraction regime, the gain in C_l, avg_ for downstroke is canceled out by an increased amount of negative lift in the upstroke. Therefore, the C_l, avg_ for the complete cycle remains negligible for all the airfoils in the propulsion flow regime as well.

B. NACA 5-Series Airfoils

In the proceeding section, the results for NACA 5-series airfoils operating in the energy extraction flow regime are presented. For NACA 5-series airfoils with reflex camber cross-section, C_d, avg_ increases 2.054 and achieves a maximum value of 2.112 as the amount of maximum camber increases as presented in Table 5. The maximum C_d, inst_ increases and achieves a maximum value of 0.114 then it starts to subsequently decrease as the amount of maximum camber is increased for airfoils having the maximum camber location near the leading-edge of the airfoils. The maximum C_d, inst_ increases as the amount of maximum camber increases for airfoils having the maximum camber location away from the leading-edge of the airfoils. C_d, avg_ increases as the location of maximum camber is moved away from the leading-edge of the airfoil. The maximum C_d, inst_ increases as the location of maximum camber is moved towards the leading-edge of the airfoil for airfoils having low maximum camber. The maximum C_d, inst_ increases then decreases for airfoils having higher maximum camber. The minimum C_d, inst_ remains negligible for all cases in this flow regime.

The maximum C_l, inst_ first increases then decreases as the amount of camber decreases when the location of maximum camber is near the leading-edge of the airfoil. The maximum C_l, inst_ increases as the amount of camber increases when the location of maximum camber is away from the leading-edge of the airfoil. The maximum C_l, inst_ first increases and then decreases as the location of maximum camber is moved away from the leading-edge of the airfoil for the airfoils with lower maximum camber. The maximum C_l, inst_ increases as the location of location of maximum camber is moved away from the leading-edge of the airfoil for the airfoils with higher maximum camber. C_l, avg_ remains negligible for all cases.

For NACA 5-series airfoils without reflex camber cross-section, C_d, avg_ increases as the amount of camber increases. The maximum C_d, inst_ increases then decreases as the amount of camber increases for airfoils having the location of maximum camber near the leading-edge of the airfoils. The maximum C_d, inst_ increases as the amount of camber increases for the airfoils having the location of maximum camber away from the leading-edge of the airfoils. The C_d, avg_ first increases then decreases when location of maximum camber is moved away from the leading-edge of the airfoil for the airfoils with lower maximum camber. C_d, avg_ increases as the location of maximum camber is moved away from the leading-edge of the airfoil for the airfoils with higher maximum camber. The maximum C_d, inst_ increases as the location of maximum camber is moved towards the leading-edge of the airfoils. The minimum C_d, inst_ remains negligible for all the cases in this flow regime.

The maximum C_l, inst_ increases as the location of maximum camber is moved towards the leading-edge of the airfoil for the cases when there is low to moderate maximum camber. The maximum C_l, inst_ increases as the location of maximum camber is moved away from the leading-edge of the airfoil for airfoils with high maximum camber. The minimum C_l, inst_ increases as the amount of maximum camber is moved away from the leading-edge of the airfoils. The maximum C_l, inst_ increases as the amount of maximum camber is decreased for airfoils with location of maximum camber near the leading-edge of the airfoils. The maximum C_l, inst_ increases as the amount of camber is increased when the location of maximum camber is away from the leading-edge of the airfoils. The minimum C_l, inst_ increases with increase with the increase in the maximum camber. C_l, avg_ remains negligible for all the cases in this flow regime.

In the following paragraphs, the results for NACA 5-series airfoils operating in the propulsion regime are presented. For NACA 5-series airfoils with reflex camber cross-section, C_d, avg_ increases as the location of maximum camber is moved towards the leading-edge of the airfoils for airfoils having moderate to high maximum cambers. The minimum C_d, inst_ increases as the location of maximum camber is moved away from the leading-edge of airfoil. As the maximum camber of the airfoils in decreased, an increase in C_d, avg_ is observed. The minimum C_d, inst_ increases as the amount of maximum camber is increased. The maximum C_d, inst_ remains negligible for all cases in this flow regime.

The maximum C_l, inst_ increases as the location of maximum camber is moved away from the leading-edge of the airfoil for airfoils having lower maximum camber. The maximum C_l, inst_ first increases then decreases as the location of camber is moved away from the leading-edge for airfoils with higher maximum camber. The minimum C_l, inst_ increases as the location of maximum camber is moved near the leading-edge of the airfoils for airfoils with lower maximum camber. The minimum C_l, inst_ increases then decreases as the location of maximum camber is moved away from the leading-edge of the airfoils for the airfoils with higher maximum camber. The minimum C_l, inst_ increases as the amount of maximum camber is decreased. C_l, avg_ remain negligible for all the airfoils in the propulsion regime.

For NACA 5-series airfoils without reflex camber cross-section, C_d, avg_ increases as the amount of camber is reduced. The minimum C_d, inst_ increases as the amount of maximum camber in increased. C_d, avg_ first increases then decreases as the location of the maximum camber is moved towards the leading-edge of the airfoils for airfoils having low maximum camber. For airfoils having more maximum camber, the C_d, avg_ increases as the maximum camber location of moved towards the leading-edge of the airfoils. The maximum and minimum C_d, inst_ increases as the location of maximum camber is moved away from the leading-edge of the airfoils.

The maximum C_l, inst_ increases as the location of maximum camber is moved towards the leading-edge of the airfoils. The minimum C_l, inst_ increases as the location of maximum camber is moved away from the leading-edge of the airfoils for airfoils having low maximum camber. The minimum C_l, inst_ increases as the location of maximum camber is moved towards the leading-edge of the airfoils for airfoils having high maximum camber. The maximum C_l, inst_ increases as the amount of maximum camber is increased. The minimum C_l, inst_ increases as the amount of maximum camber is decreased. C_l, avg_ remains negligible for all airfoils in this regime.

**Fig. 12:**
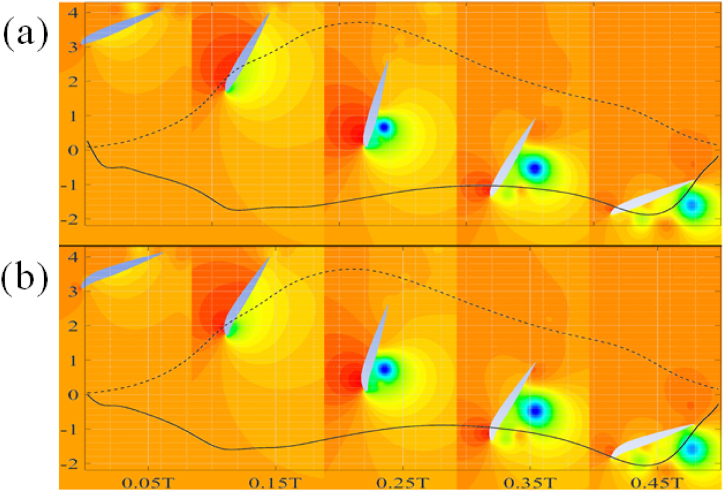
Airfoils undergoing downstroke motion. (a) NACA 22010, (b) NACA 54010.

**Fig. 13:**
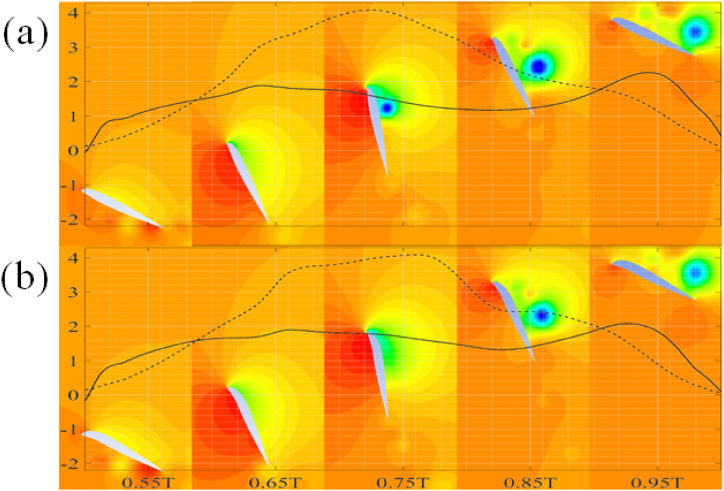
Airfoils undergoing upstroke motion. (a) NACA 22010, (b) NACA 54010.

The observed trends reported in Table 5, Table 6 are further explained using the pressure contours for flapping motion of NACA 22010 and 54010 airfoils. The downstroke at various time instances is shown in Fig. 12(a) and (b) while the upstroke is shown in Fig. 13(a) and (b). Within Fig. 12, Fig. 13, the dashed line, “--”, represents C_d, inst_ while the solid line “__” represents C_l, inst_. It is observed that there is negligible difference between C_d, inst_ during the downstroke between the two airfoil types. The C_d, avg_ for downstroke is 1.94 for NACA 54010 as compared to 1.92 for NACA 22010. This negligible difference in C_d, avg_ can be explained by almost the same pressure values at the aft and fore of the airfoils. The NACA 22010 is observed to have slightly more negative C_l, inst_ between ∼0.1T and ∼0.3T. The more negative lift is due to the lower pressure at the leading-edge of the airfoil which in turn is due to the formation of the leading-edge vortex. The vortex formation takes place earlier in the cycle for airfoils with low maximum camber and location of camber near the leading-edge of the airfoils. As a result, the C_l, avg_ for the downstroke is around 3 % more NACA 22010 as compared to NACA 54010. The C_l, avg_ comes out to be −1.174 for NACA 54010 as compared to −1.214 for NACA 22010. The C_d, avg_ for NACA 54010 during upstroke comes out to be 2.236 as compared to 2.183. The higher C_d, avg_ is due to higher pressure difference between aft and fore of the NACA 54010 as compared to NACA 22010, specially at ∼0.55–0.65T and ∼0.75T–0.9T, as visible in Fig. 13. At ∼0.55T for example, the higher pressure in the concave region near the leading-edge, on the pressure side of the NACA 54010 airfoil is the cause for higher C_d, avg_. Meanwhile, the relatively lower pressure in the wake of the NACA 54010 airfoil is responsible for a higher C_d, avg_ at ∼0.85T. The NACA 54010 has more positive C_l, inst_ in the at ∼0.68–0.91T. This can be explained by the lower pressure at the leading-edge of the NACA 54010 airfoil, caused by the leading-edge vortex as the vortex sheds. The C_d, avg_ during upstroke for NACA 54010 comes out to be 1.503 as compared to 1.462 for the NACA 22010. It is to be noted that the in the contours shown throughout the manuscript, the red color represents maximum and dark blue color represents the minimum observed values.

C. Reflex-Camber and VS No Reflex-Camber Airfoils, A Comparison

**Fig. 14:**
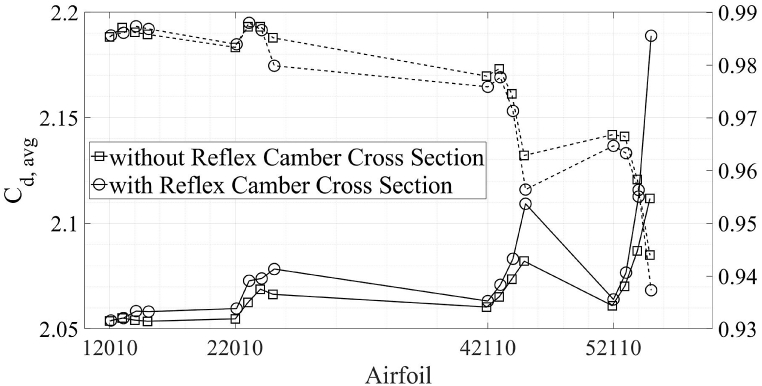
Vertical axis L-R; C_d, avg_ Energy Extraction, C_d, avg_ Propulsion.

**Fig. 15:**
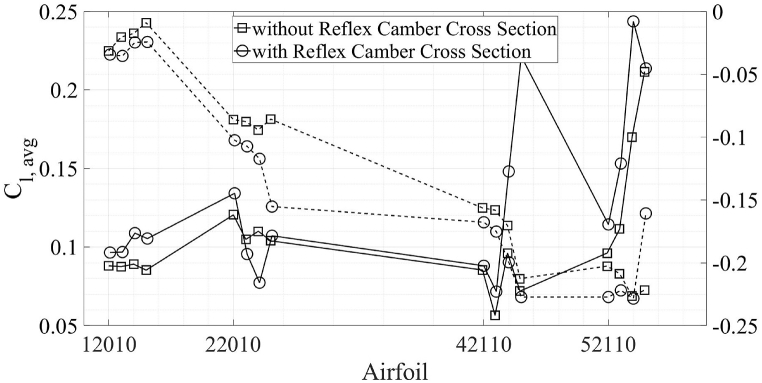
Vertical axis L-R; C_l, avg_ Energy Extraction, C_l, avg_ Propulsion.

**Fig. 16:**
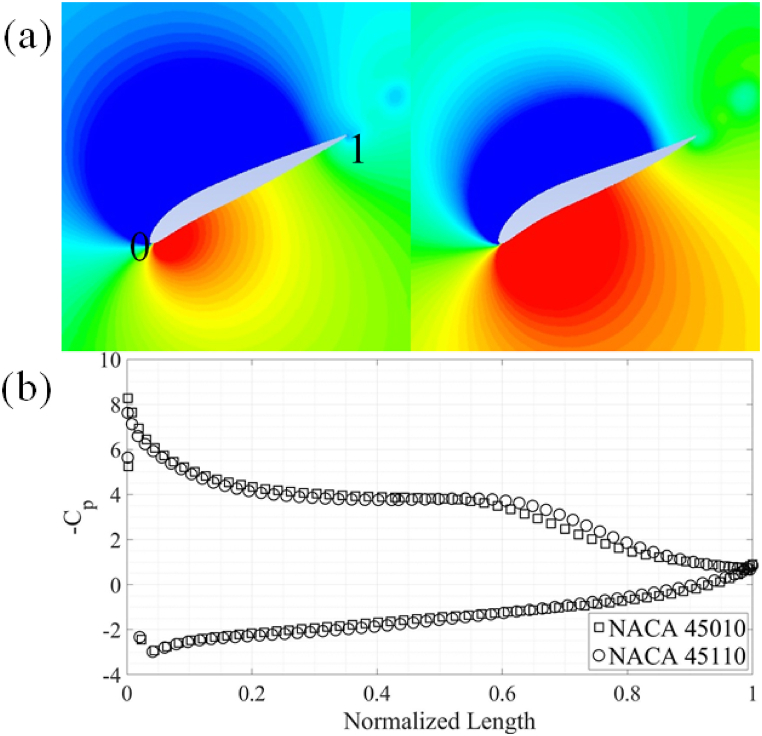
(a) Pressure contours around the NACA 45010 and NACA 45110 airfoils. (b) Comparison of surface C_P_.

**Fig. 17:**
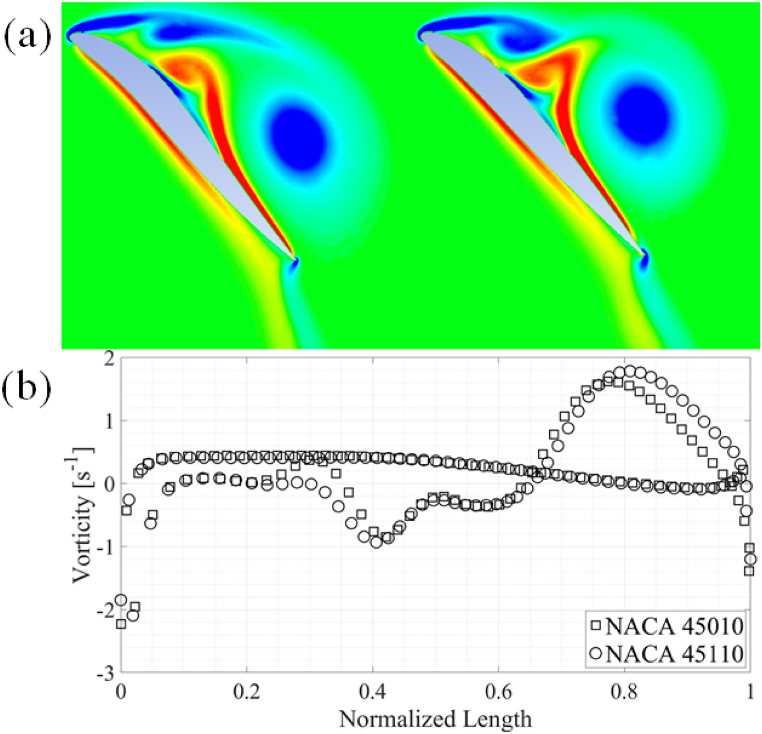
(a) Vorticity around NACA 45010 and NACA 45110 airfoils. **(**b**)** Comparison of vorticity.

In this section, a comparison between airfoils with no reflex camber and reflex camber is presented. Fig. 14, Fig. 15 shows a comparison of C_d, avg_ and C_l, avg_ between airfoils with and without reflex, respectively. It is clear from Fig. 14 that airfoils with reflex camber have slightly more C_d, avg_ in the energy extraction regime as compared to the airfoils without reflex camber and vice versa. On the other hand, Fig. 15 shows that airfoils with reflex camber produce more lift in both the energy extraction and propulsion regimes. It should be noted that the C_d, avg_ and C_l, avg_ values for the propulsion regime are multiplied by negative one to avoid clutter in Fig. 14, Fig. 15. Furthermore, within Fig. 14, Fig. 15, dashed lines, “--”, indicate propulsion flow regime while the solid lines indicate energy extraction regime. The underlying flow physics for these trends is further explained by using the vorticity and coefficient of pressure contour plots in Fig. 16, Fig. 17 in the proceeding paragraphs.

The observed trends of maximum and average force coefficients are elaborated using comparison of the results for NACA 45110 and 45010 airfoils. The results can be explained by closely looking at the C_p_ plots at 0.8T as shown in Fig. 16. The pressure contours are also shown in Fig. 16(a) and (b). There is a relatively large pressure difference between the leading- and the trailing-edge of the NACA 45010 airfoil as compared to the NACA 45110 airfoil, resulting in more C_d, avg_ in the airfoil without reflex camber. It is also evident from the C_p_ plot and as well as from the pressure contours that a larger pressure difference exists between the suction and pressure sides of the NACA 45110 as compared to NACA 45010, resulting in higher C_l, avg_ for NACA 45110. This pressure difference is more evident from mid-chord towards the trailing-edge of the airfoils. It is also noticed that the higher-pressure region is confined within the concave region on the pressure side of the airfoil with reflex camber cross-section.

Furthermore, it is evident from Fig. 17(a) and (b) that stronger vortex formation takes place at the leading-edge of the airfoil with reflex camber cross-section. This vortex results in the lower pressure zones in the wake of the NACA 45110 airfoil. It should also be noted that due to a higher curvature on the pressure side of the airfoils with reflex camber cross-section, a relatively higher pressure is developed on the pressure side of the airfoils with reflex camber. This along with lower pressure due to the stronger leading-edge vortex contributes to a higher C_d, avg_ and C_l, avg_ for airfoils with reflex camber.

## 4 Conclusion

The main objective of the present research is to conduct a systematic study to analyze the effect of the airfoil shape on the aerodynamics forces for the flapping motion of an airfoil. By analyzing the results, it can be clearly seen that airfoils with high maximum camber located near the leading-edge of the airfoils generally have maximum drag and lift for energy extraction regime. For NACA 4-series airfoils operating in the propulsion regime, a trade-off is noticed between C_l, avg_ and C_d, avg_. Generally, airfoils with maximum camber location near the leading-edge of the airfoil have the most C_l, avg_ and C_d, avg_, while the airfoils with higher camber have more C_l, avg_ and vice-versa. For airfoils operating in the energy extraction regime, the airfoil having the most C_d, avg_ is identified as NACA 8210. Meanwhile, the NACA 4-series airfoil with most C_l, avg_ is the NACA 6210. For NACA 4-series airfoils operating in the propulsion regime, the airfoil having the most C_d, avg_ is identified as the NACA 2210 while the airfoil with most C_l, avg_ is identified as the NACA 8210. Therefore, the NACA 8210 can be described as the best performing NACA 4-series airfoil, in terms of aerodynamics forces. For NACA 5-series airfoils, it is found that a higher C_l, avg_ is shown by airfoils with a high maximum camber and location of the maximum camber located away from the leading-edge of the airfoils for both flow regimes. This phenomenon also holds true for C_d, avg_ for airfoils operating in the energy extraction regime. For the propulsion regime, a higher C_d, avg_ value is achieved by airfoils with low maximum camber, having the maximum camber near the leading-edge of the airfoils. For the NACA 5-series airfoil without reflex camber cross-section, operating in the energy extraction flow regime with most C_d, avg_ and C_l, avg_ is shown by 55010. For the NACA 5-series airfoil with reflex camber cross-section, the most C_d, avg_ is 55110 and the most C_l, avg_ is shown by 54110. For the NACA 5-series airfoil without reflex camber cross-section, operating in the propulsion regime, most C_d, avg_ is shown by 24010 and the most C_l, avg_ is shown by 54010. For the NACA 5-series airfoil with reflex camber cross-section, the most C_d, avg_ is 23110 and the most C_l, avg_ is shown by 54110. This study will facilitate the selection of the best airfoil for the development of a hydrokinetic turbine and a micro air vehicle. The research work on these projects in on going.

## Funding

This research was funded by the Researchers Supporting Project number RSP2024R373.

## Data availability statement

The data required to reproduce the results of the research work carried out in this paper are included in article.

## CRediT authorship contribution statement

**Fahad Butt:** Formal analysis, Data curation, Conceptualization. **Tariq Talha:** Supervision, Formal analysis, Data curation, Conceptualization. **Rehan Khan:** Project administration, Methodology, Investigation. **Abdur Rehman Mazhar:** Writing – review & editing, Visualization, Validation. **Mahad Butt:** Writing – review & editing, Validation, Software. **Jana Petru:** Visualization, Resources, Investigation. **Asiful H. Seikh:** Writing – review & editing, Visualization, Data curation.

## Declaration of competing interest

The authors declare that they have no known competing financial interests or personal relationships that could have appeared to influence the work reported in this paper.
